# Polymer-Based Constructs for Flexor Tendon Repair: A Review

**DOI:** 10.3390/polym14050867

**Published:** 2022-02-23

**Authors:** Jef Brebels, Arn Mignon

**Affiliations:** Surface and Interface Engineered Materials, Campus Group T, KU Leuven, Andreas Vesaliusstraat 13, 3000 Leuven, Belgium; jef.brebels@kuleuven.be

**Keywords:** flexor tendon repair, anti-inflammatory, anti-adhesion, antimicrobial, polymer-based constructs

## Abstract

A flexor tendon injury is acquired fast and is common for athletes, construction workers, and military personnel among others, treated in the emergency department. However, the healing of injured flexor tendons is stretched over a long period of up to 12 weeks, therefore, remaining a significant clinical problem. Postoperative complications, arising after traditional tendon repair strategies, include adhesion and tendon scar tissue formation, insufficient mechanical strength for early active mobilization, and infections. Various researchers have tried to develop innovative strategies for developing a polymer-based construct that minimalizes these postoperative complications, yet none are routinely used in clinical practice. Understanding the role such constructs play in tendon repair should enable a more targeted approach. This review mainly describes the polymer-based constructs that show promising results in solving these complications, in the hope that one day these will be used as a routine practice in flexor tendon repair, increasing the well-being of the patients. In addition, the review also focuses on the incorporation of active compounds in these constructs, to provide an enhanced healing environment for the flexor tendon.

## 1. Introduction

The flexor digitorum superficialis, or in short, the flexor tendon, is an irreplaceable part of the human body. Connecting muscle to bone provides strength and stability, the ability to withstand tension, transmit forces, and release stored energy. Since tendons are subjected to repeated motions and degeneration over time, they are vulnerable to acute and chronic injuries [1,2,3]. Hand tendon traumas comprise approximately 10% of all emergency department visits and up to 20% of all injuries treated [4,5]. Athletes, construction workers, military personnel, and others who make repetitive movements have a greater risk of injuring the flexor tendon by tearing or rupturing. A trauma impact directly to the hand could also lead to such an injury. Injured flexor tendons will exhibit a biological attempt to heal the inflicted damage. However, the speed at which this happens is greatly outpaced by the own capacity of accumulating further damage. Therefore, it should be noted that flexor tendons cannot undergo spontaneous healing and operational procedures are almost always required [6,7]. In addition, flexor tendons have an extended healing period of up to 12 weeks due to their limited blood flow and hypocellularity [8,9].

So far, multiple therapeutic reconstruction techniques such as suturing, auto-, allo-, and xenograft or replacement with a synthetic prosthesis have been used [10,11]. Unfortunately, none of these traditional techniques accomplish a long-term adequate solution for postoperative complications such as infection, wear, tendon scar tissue formation, mechanical failure, and excessive adhesion formation [12]. The success and effectiveness of these traditional repair techniques are mostly linked to the degree of undesired postoperative adhesion formation between surrounding tissue and the healing site [7]. It is important to note that the original mechanical properties are never fully restored after tendon repair due to scar tissue formation around the healing site. The scar tissue is inferior in mechanical properties due to the predominant presence of type III collagen, whereas healthy tendon tissue mainly consists of type I collagen. An excessive amount of type III collagen results in loosely organized fibrils [13]. These complications can be avoided by inducing a healing response that is faster than the rate of adhesion and scar tissue formation [14].

New treatment strategies have emerged to overcome these clinical challenges. Tissue engineering for flexor tendon repair by combining cells and growth factors on interactive scaffolds formed the next promising repair technique [15]. Tissue engineering has gained popularity in the field of regenerative medicine due to its bioactivity and biocompatibility. The scaffold is used as a biomaterial that enables critical functions such as cell adhesion, proliferation, cell-biomaterial responses, and cell differentiation in the body. Scaffold vascularization is often a problem occurring in tissue engineering. The supply of oxygen through the scaffold to the surrounding tissue is essential for maintaining cellular respiration. Additionally, cellular functions including proliferation and differentiation are only possible when essential nutrient exchange and removal of toxins and waste products from the scaffold is ensured [16]. The scaffold must be able to achieve these functions without inducing an immune reaction [17]. However, compared to the traditional solutions, similar problems, as described above, appear such as the lack of mechanical strength in vivo which is needed for flexor tendon repair since they support large mechanical stresses [18]. Therefore, an alternative route of current experimental research is more focused on producing a material-based mechanical construct that is placed around the damaged tendon area. The construct acts as a mechanical and physical barrier to minimize the formed adhesion, without compromising the diffusion of nutrients and by-products produced by the biodegradation of the construct [19]. The ideal construct should provide sufficient mechanical support as well as provide the tendon with a controlled environment to regrow and if needed reattach. This review article acts as a literature scan to identify the current state-of-the-art research for flexor tendon repair constructs, some of which have active compounds incorporated which induce anti-inflammatory and antimicrobial properties.

## 2. Flexor Tendon

### 2.1. Flexor Tendon and Function: Composition and Structure

The flexor tendon is composed of dense, fibrous, and highly organized tissue responsible for the connections between muscle and bone. Their function is to transmit the forces produced by muscle contraction to the skeletal bones, making body motion possible [1,2]. Other functionalities include the resistance against large tensions, storage, and release of energy, providing strength, and ultimately, stability, to the fingers [3]. Healthy tendons allow for great resistance to mechanical stress with minimal deformation due to their fibro-elastic texture [20,21].

Flexor tendon functionality is closely linked to the composition of the extracellular matrix (ECM). The major component of the flexor tendon is water (60–80%) whereas type I collagen makes up for approximately 70–80% of dry weight. Other types of collagens (III, V), proteoglycans, and glycoproteins such as mainly elastin account for the remainder of 20–30% of dry weight. Elastin in combination with type I collagen is responsible for flexibility and strength respectively [21,22,23].

**Figure 1:**
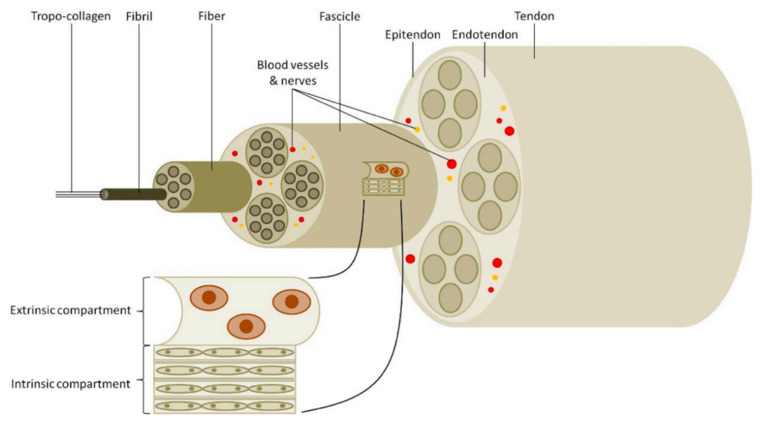
Hierarchical structure of the tendon. Collagen molecules are shown in the simplified model of the tendon structure to represent the forming complex arrangement from tropo-collagen up to tendon fascicles and the final tendon tissue. The intrinsic compartment is represented by the tendon fascicles as the basic unit. The extrinsic compartment is represented by the synovium-like tissue connecting the vascular, nervous, and immune systems. Reprinted with permission from ref. [12]. 2020 Angelo V. Vasiliadis.

The aggregation of type I collagen molecules forms a collagen fibril. These collagen fibrils bundle into fibers containing fibroblast cells, fibers further bundle into fascicles, and finally, fascicles bundle together to form the fascicular matrix. The endotenon occupies the small space between the fascicle bundles. The fascicular bundles can make small slip motions due to the endotenon containing limited nerves, blood, and lymphatic vessels. All fascicles group together and are surrounded by yet another connective tissue sheath, the epitenon [21,24,25,26,27]. The hierarchical structure of the tendon can be seen in Figure 1. Assemblies of type I collagen in fibrils, fibers, and fascicles exhibit a wave pattern, also known as crimp, that will align and straighten when a tensile loading is applied [28]. They also act as a shock absorber giving the tendon the possibility to absorb and transmit the applied tension forces, avoiding possible tissue damage.

**Figure 2:**
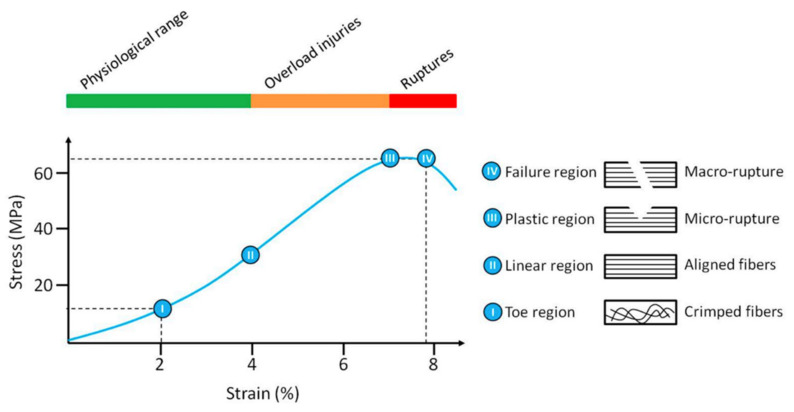
Typical stress-strain curve for a healthy tendon. The righthand side is a schematic representation of the mechanical behavior of the collagen fibers for the different regions. The physiological range (green) consists of the region with the normal use of the tendon and is followed by the overload injuries region (orange) where permanent damage occurs, starting with microscopic failure. Further strain of the tendon will lead to the failure region (red) where rupture of the tendon takes place. Reprinted with permission from ref. [12]. 2020 Angelo V. Vasiliadis.

As mentioned above, the functionality of flexor tendons is closely linked with the composition and structure of the ECM, responsible for a non-linear viscoelastic and anisotropic behavior, making tendons capable of withstanding high-tension loads [21]. This unique mechanical behavior can be reflected in four distinct regions of the stress-strain curve including the toe, linear, plastic, and finally the failure region [26] (See Figure 2). Tendons also exhibit a viscoelastic behavior, meaning the stress-strain response of the flexor tendon is strain rate dependent. Stretching of the tendon at higher strain rates results in less deformation and higher stiffness while maintaining the same regions during the damage process [29,30]. Therefore, tendons loaded at low strain rates will tend to absorb more energy, although they are less effective in withstanding mechanical stress. “Un-crimping” happens in the toe region for tensile loads up to 2% strain. The slope of the toe region is not constant, resulting in a non-linear stress-strain curve. Once all collagen fibrils have been aligned (2% strain), the slope becomes linear and Young’s modulus can be determined. The collagen fibrils stretch physically due to intermolecular sliding of the collagen triple helices [31] (2–4% strain). Tendons are elastically deformed until this point and will still return to their original state and length once the tensile load is removed. Additional loading, thus for a strain >4%, causes microscopic failure to individual fibrils which are irreversible plastic deformation. The tendon is stretched beyond the physiological limit, initiating macroscopic failure followed by failure of the whole tendon when strains are reaching higher values than 8–10% [32,33,34]. Partly overloading the tendons before macroscopic failure or providing insufficient recovery time can cause permanent damage to the collagen fibrils, lowering the functionality of the tendon [2,35]. Fatigue loading also has a significant influence on the structure of the tendon, permanently altering the crimp characteristics [28].

### 2.2. Flexor Tendon and Function: Causes and Impact

Since tendons are subjected to repeated motions and degeneration over time, they are vulnerable to acute and chronic injuries, direct or indirect. Direct acute tendon injuries occur because of sports activities, blunt impact trauma, or lacerations by a sharp object, accompanied by a loss of tensile strength and disarrangement or possible rupture of the tendon bundles. Tensile overloading, repetitive microscopic failure (fatigue included) or intratendinous degeneration due to aging will lead to chronic indirect flexor tendon injuries. Most commonly, these indirect injuries will occur at the junctions (osteotendinous or musculotendinous) because a healthy tendon can withstand higher tensile stress in the central part of the tendon, compared to the junctions [36]. Tendinopathy, affecting approximately 100 million people worldwide annually, is a failed healing response of the tendon, characterized by swelling, hindered performance, and pain [2]. Interestingly, for decades, researchers believed tendinopathy to be non-inflammatory [37]. Nonetheless, more recent research on tendinopathy confirms the inrush of inflammatory cells, including macrophages and lymphocytes. In addition, it has been hypothesized that these inflammatory cells play a significant role in the early initiation of tendon pathologies. Various mediators of inflammation, pro-inflammatory cytokines, are proven to be expressed during tendon injury such as; Tumor Necrosis Factor-α (TNF-α), Interleukin-1 β (IL-1 β), and Interleukin-6 (IL-6) [38,39]. Even minor injuries may result in a significant high economic, social, and clinical impact on society and patients due to lost work time and high socioeconomic costs [40].

### 2.3. Flexor Tendon and Function: Regeneration and Repair

Tendons have a weak intrinsic healing ability due to their hypocellularity and hypovascularity structure. This problem, in combination with postoperative complications, makes for inefficient and prolonged healing [41]. Damaged flexor tendons tend to heal after injury, although original mechanical and biological properties are never completely restored due to the higher ratio of type III collagen to type I collagen in the collagen fibers after repair [42]. This increases the risk of degeneration and re-rupture at the repaired injury site along with functional impairment. Lower mechanical properties result in a decrease in grip strength of the fingers and hand. Literature [43] indicates a mean grip strength of 74.5% compared to the opposite uninjured hand.

The healing mechanism of tendons is divided into intrinsic and extrinsic healing operating either independently or in cooperation with one another. Tenocytes and fibrocytes at the injury site will result in intrinsic healing, while extrinsic healing is a result of the invasion and proliferation of fibroblasts and inflammatory cells from surrounding tissue and tendon sheath in order to produce a new collagen matrix. The degree of involvement of these external cells is dependent on the vascular perfusion to the injury site. Commonly, these external cells will predominate over the resident cells, causing the surrounding tissue to attach to the repair site, with cell adhesion as an adverse effect. Sufficient vascularity and nutrition by surrounding fluids, absence of cell adhesion, and resident cells proliferation are depending on factors of intrinsic healing [44,45,46,47,48,49].

**Figure 3:**
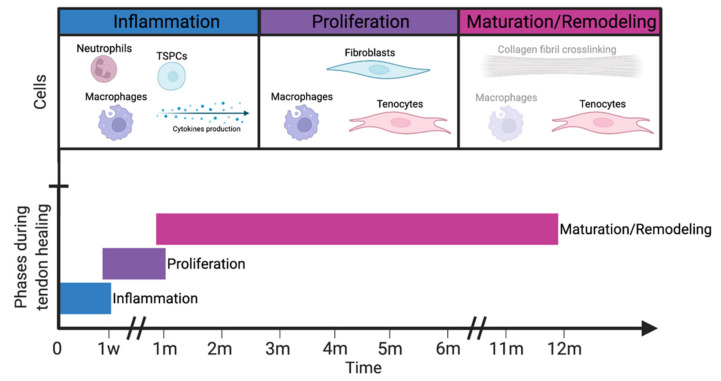
Overview of the process during tendon healing. Healing includes three phases, which overlap slightly. The duration of each phase is an estimate, as duration depends upon the location and severity of the tendon injury.

Tendon healing after surgical repair follows three well-described, consecutive but overlapping phases: (i) inflammation, (ii) proliferation, and (iii) remodeling [50], represented in Figure 3. The inflammation phase is rather short, which lasts only approximately a week, followed by the proliferation phase, lasting up to 4 weeks. The last phase, remodeling, takes several months to complete [51]. During the inflammation phase, immediately after injury, vascular permeability increases, and inflammatory cells infiltrate the healing site on the tendon (forming a hematoma). These cells include macrophages, monocytes, neutrophils, and tendon stem/progenitor cells (TSPCs). These latter produce cytokines like IL-6 and IL-1 β, responsible for regulating the immune responses by attracting fibroblasts to the injury site [37].

Neutrophils, released into the blood vessels, contribute to the healing process by removing foreign cells and strengthening the inflammatory response. Macrophages, in addition, are essential in both promoting and resolving the inflammatory response as well as both moderating and facilitating tissue repair [49]. They remove dead cells and toxic metabolites from the injury site by phagocytosis. TSPCs differentiate into tendon-like cells to play an essential role in the whole healing process. The inflammation phase is followed and partly overlapped, by the proliferation phase as can be seen in Figure 3 [36]. During proliferation, growth factors, including basic fibroblast growth factor (bFGF), bone morphogenetic proteins-12, -13, and -14 (BMPs) also known as growth and differentiation factors-5, -6, and -7 (GDFs) respectively, transforming growth factor-beta (TGF-β), insulin-like growth factor-1 (IGF-1), platelet-derived growth factor (PDGF) and vascular endothelial growth factor (VEGF) released by macrophages and endothelial cells which are involved during different phases of the healing and regeneration process [52]. Next, recruited fibroblasts (by cytokines) synthesize components of the ECM; mostly type III collagen, proteoglycans, and fibronectin. The newly formed ECM is randomly arranged and forms a bridge between the injured regions. Lastly, the remodeling phase (see Figure 3) can be divided into two, consolidation and maturation. Consolidation begins 6–8 weeks after the injury and can take up to 10 weeks to complete. Here, a decrease in cellularity and ECM production is observed. The tissue becomes more fibrous due to the replacement of randomly organized type III collagen with crimp-oriented type I collagen. The organization of type I collagen fibers to the longitudinal axis partly restores the tendon tensile strength and stiffness [45,53]. The maturation starts after approximately 10 to 12 weeks, characterized by an increase in collagen fibril crosslinking and further conversion of type III to type I collagen. An important point to note is that the tendon is considered “healed” after a maximum of 12 weeks. This means that the finger can be used again for normal movements. However, the maturation process will continue in the background to increase the mechanical properties of the tendon. Finally, more mature tendonous tissue is formed [36,54].

## 3. Traditional Strategies for the Repair of Flexor Tendon Injuries

Lacerated tendons do not have the ability to repair themselves without surgical intervention. Tension in the flexor tendon causes the injured end to retract up to several centimeters into the palm of the hand [55,56]. Ultrasound and magnetic resonance imaging can operate as a tool in order to locate the retracted flexor tendon end pre-operatively, although they are not routinely used [57,58]. Surgical repair is necessary to regain the finger mobility, lost after injury. In most cases, flexor tendon surgery is performed in the emergency department’s operating room under general or local anesthesia. A flexor tendon injury is commonly accompanied by fracture and loss of the skin, nerve lacerations, and damage to the blood vessels [59]. All these injuries, along with the tendon injury, need to be repaired during the same operation. There are a variety of operative measures available for repairing the flexor tendon in humans, with suture and graft transplantation techniques being the two most commonly used.

### 3.1. Suture Techniques

Clinically, a lot of different suture techniques are used such as Lin locking, Kessler, Modified Kessler, Savage, Becker, Tajima, grasping, and epitendinous suture [36,60]. The following two are widely accepted: (i) the Modified Kessler suture utilizing 2 sutures [61] and (ii) the grasping suture [62] which uses knots located in the cross-section. Past research has proven that the number of core strands crossing the repair site is directly proportional to the strength of the tendon repair, whereas most ruptures occur at the knots [59,63,64,65]. However, an increased number of core strands will at the same time increase the complexity and tendon gliding resistance [11]. It is recommended to use a core suture purchase of 7 to 10 mm, as it provides higher gap strength and resistance [59].

In addition to the various possible techniques, several studies have been conducted on the influence of the suture material as well. Some of the commonly used non- adsorbable materials for sutures include monofilament nylon, braided polyester, and monofilament poly(propene). Nylon has the lowest ultimate tensile strength, followed by polypropylene and polyester respectively [66]. Both adsorbable and non-adsorbable materials are capable of early active rehabilitation along with similar rupture rates and mechanical outcomes [11,56,59,66]. Another material used in more recent research is monofilament stainless steel, characterized by its increased stiffness and strength compared to the other suture materials [67]. Reoperation is necessary when non- adsorbable threads are used because they are unaffected by the biological activities of the body tissues, which could increase the chance of infections and scar formation. Finally, the ideal suture should also respond minimal to surrounding tissue and be easily manipulated [68].

A meta-analysis recently performed revealed rates of 4% rupture, 6% re-operation, and 4% adhesions after operative suture repair. In addition, studies showed no difference in rupture rate when core strand or epitendinous suture technique was used. The same study also showed a decrease of 84% in re-operation when the epitendinous suture was used and adhesion formation decreases by 57% with the modified Kessler suture technique [69]. Rupture of the repaired tendon happens most commonly 1 or 2 weeks after repair due to the weakness of the repair site, overaggressive therapy, or non-compliance of the patient [70]. There is a lot of room for improvement despite the recent advances in clinical research. Reoperation is often required, and a significant adhesion formation is still present, these two being the main complications of the suture technique.

### 3.2. Graft Transplantation Techniques

The suture technique is seen as the primary repair method for flexor tendon injuries but is not always possible due to infections or when part of the tendon is non-viable. Grafting can be utilized in these cases to bridge the gap and restore the flexor tendon. It can be seen as a secondary reconstruction method when the primary suture technique has failed. Satisfactory results have been obtained by this procedure providing there was no damage to the digital sheath and the muscle was still intact [71].

Auto- (same species, own body) and allografts (same species, different body) are the two most used grafting techniques to repair flexor tendons. The palmaris longus, the plantaris, and sometimes the toe extensor include the most suited tendons used as grafts [72]. Allografts are limited in supply and may cause the transmission of diseases and increased inflammatory reactions to the donor cell tissue. In addition, a decrease in tensile properties has been observed for allografts that have been irradiated or chemically sterilized [73,74]. Autografts are less limited in supply although significant donor-site morbidity has been observed, causing tendonitis, pain, and muscle deterioration [75]. Unfortunately, identical to suture procedures, flexor tendon grafting procedures will also cause the formation of adhesion with all the negative consequences previously described. Although the mechanism of tendon graft repair is poorly understood, adhesion formation with autografts is believed to arise from intrinsic (tenocyte necrosis) and extrinsic fibrosis (influx of synovial and inflammatory cells), leading to excessive scarring [59,76,77]. Other research reported minimal formation of adhesion using acellular allografts on bovine flexor tendon repair [78,79] indicating that acellular allografts can heal without the intrinsic healing mechanism, often observed in autograft tissue.

In contrast to auto- and allografts, xenografts do not originate from the same species. Xenografts have higher immunogenicity than allografts, causing rejection by the host to be the most important limitation [10,80,81]. Similar to allografts, there is a risk of disease transmission from donor to host. The risk of disease transfer is higher when using xenografts due to the fact that there are several zoonotic diseases that are unknown and could pose a great risk to the patient and society [80,82]. A high postoperative infection percentage of 20.6% was observed in the literature where immunochemically modified porcine patellar tendon xenografts were used to reconstruct human anterior cruciate ligament (ACL) tissue [83]. Therefore, allo- and xenografts are often decellularized to reduce immunogenicity. Several processes have been used like cell rinsing, freeze-drying, sterilization, etc. These two types of grafts are as a result non-viable in nature and will act more as inert pieces in the body [84]. This causes the allo- and xenografts to incorporate significantly worse with the injured tissue resulting in rapid adsorption of the graft during healing [85].

James Hunter (1960s–1980s) was among the first to describe refined techniques of using artificial tendons as grafts, along with artificial gliding, and implants to reconstruct the tendon sheath [86,87]. Synthetic tendon grafts do not require graft harvesting from a donor or donor-site, shortening the surgical procedure. In addition, autografts often experience a necrotic stage before the prolonged healing stage, which is not the case for synthetic grafts. Finally, synthetic grafts have similar mechanical properties and dimensions with abundant availability compared to the other three earlier described graft types. Artificial tendon grafts clinically available include carbon (Johnson and Johnson, New Brunswick, NJ, USA), carbon and polyester (Surgicraft, Nairobi, Kenya), Dacron (Stryker-Meadox), Leeds-Keio polyester (Neoligaments, Yeadon, UK), and Gore-Tex polyctetrafluoroethylene) (WL Gore, Hong Kong, China) [88]. The usage of these synthetic grafts has declined over the years due to many problems. Tissue reaction from debris particulates may cause pyogenic flexor tenosynovitis (PFT), characterized by inflammation of the fluid-filled sheath, after 4–5 years [89,90]. In addition, synthetic grafts often have a high failure rate and poorer outcomes. Finally, they also significantly increase the cost of the operation; artificial ligaments are costly [91].

## 4. New Strategies for the Repair of Flexor Tendon Injuries

The traditional strategies for flexor tendon repair can lead to serious complications, as mentioned above, despite the positive outcomes in the short term. The need of solving the shortcomings of traditional strategies for flexor tendon repair techniques has prompted the research of alternatives such as construct designs from polymeric materials that wrap around the injured tendon, not to be confused with synthetic grafts which replace the injured tendon.

### 4.1. Biochemical Solutions for Postoperative Complications

Research has shown that several factors affect flexor tendon healing and cell adhesion formation due to the invasion of external fibroblasts. The formation of post-surgical scar tissue and cell adhesion between surrounding tissue and tendon constricts tendon gliding and motion, causing a loss in functionality. In some situations, although they are rare, infections occur after flexor tendon repair [49]. These postoperative complications are still major clinical challenges to overcome.

#### 4.1.1. Peritendinous Adhesion Formation

Adhesion formation can be minimized or even prevented by optimizing the intrinsic healing mechanism. Past researchers believed that flexor tendon healing was strongly dependent on extrinsic cellular ingrowth, which relies on adhesion formation at the site of injury. However, it was documented that flexor tendons should have the ability to heal by intrinsic healing mechanisms alone [48]. Intrinsic healing can be optimized by using biochemical factors to achieve scarless healing. Current intrinsic healing optimization methods include physical and mechanical barriers to prevent adhesion formation as well as chemical and molecular compound addition against scar tissue formation [92]. Ideally, physical barriers are combined with chemical/biological modulation to produce a superior biomaterial construct to prevent peritendinous adhesion.

Physical barriers form the first method for the prevention of peritendinous adhesion formation. Placing an anti-adhesive material, acting as a barrier, between the healing site and the surrounding tissue limits the contact between the tendon injury site and its sheath, diminishing the amount of surface available for adhesion formation. Hereby, the tendon is restricted to intrinsic healing, healing only to itself and not to surrounding tissue or the tendon sheath [14].

Other researchers have made use of the therapeutic properties of biological polymers in combination with synthetic polymers to form semi-synthetic materials as an attempt to minimize the formation of scar tissue and peritendinous adhesion. The ideal chemical compound should be limited to a single application, have no systemic side effects, and should target immediate inflammatory responses and the extrinsic healing mechanism. A decrease in inflammation is accompanied by a decrease of invasive external cells to the injured site of the tendon, minimizing the scar tissue and peritendinous adhesion. The anti-inflammation character of these chemical agents is thus responsible for the reduction of adhesion formation. Chemical compounds used in recent research include nonsteroidal anti-inflammatory drugs (NSAID’s) like naproxen [93], ibuprofen [94,95,96,97,98], 5-fluorouracil [99], and celecoxib [100,101,102], steroids like dexamethasone [103] and other polymers such as hyaluronic acid (HA) [93,100,102,104,105,106,107,108] and chitosan [19,109] (Figure 3, see Section 4.3). The common function of these chemical agents is the reduction of the inflammatory response and consecutively the reduction of adhesion.

Another key aspect in the prevention of peritendinous adhesion after an operation is the early mobilization of the injured finger, as this will cause the tendon to glide, which will ensure adhesion formation is minimized. Historically, fingers were immobilized after flexor tendon repair using an external brace until the tendon healed sufficiently before allowing movement [110]. Hereby, adhesion formation could not be prevented leading to the decrease in functionality of the finger. New strategies of early passive postoperative mobilization have been developed in the late 1970s to promote tendon gliding. Early passive mobilization decreased adhesion formation and increased the tensile strength at the injured site [111], but might also cause deformation of the tendon [7]. Early active mobilization immediately after a repair has been recognized as an important treatment for enhancing flexor tendon healing [112]. Strong material-based constructs are needed for tolerating the forces of the early active movements, as the construct is solely responsible for the strength of the repair during the early days after operating [113]. However, this technique is also accompanied by an increase in ruptures of the load-bearing constructs due to the difficulty in controlling the loads from active movement [114,115].

#### 4.1.2. Infections

Infection is most commonly caused by a significant degree of contamination during the initial injury [49]. Infection rates increase when the trauma was caused by for example maritime or agricultural activities. In addition, infections depend on the type of injury with increased infection rates for bite wounds, crush injuries, replantation, and injuries with accompanied fractures [116,117,118]. In 2018, a review paper [119] reported the most found microbial populations causing flexor tendon infections such as i.e., *Streptococcus pyogenes*, *Mycobacterium tuberculosis*, *Mycobacterium tuberculosis*, *Staphylococcus*, and many more with the latter being the most frequently isolated bacterium. Flexor tendon sheath infections can have a devastating effect leading to morbidity and even the loss of a finger. Therefore, it is important to address the presence of such contamination or early infection prior to the surgical procedure. Good results have been obtained using closed tendon sheath irrigation, antibiotics, and debridement [120]. However, patients with severe PFT are still at risk of morbidity. Leaving PFT untreated will result in rapid deterioration of the gliding mechanism and will cause adhesion formation. Fast observation and treatment of PFT are essential to prevent the disruption of finger and hand functionality. A physical examination is needed in order to identify Kanavel’s four cardinal signs of infections as follows: (i) a finger held in slight flexion, (ii) fusiform swelling of the affected digit, (iii) tenderness along the flexor tendon sheath, and (iv) pain with passive extension of the digit [121,122]. Recent development indicates that it is possible to integrate an antimicrobial compound, such as silver nanoparticles (Ag NPs) [68,104,123,124,125], for example, into material-based constructs used for tendon repair in order to mitigate the infection risk.

### 4.2. Requirements of Polymeric Materials for Flexor Tendon Repair

Several polymeric (bio)materials have been explored as alternatives to the traditional repair strategies. Neighboring fields of cartilage and bone tissue engineering formed an inspiration for possible polymer-based constructs. In addition, some have been specifically developed to mimic the ECM and biomechanical structure of the flexor tendon. The ideal polymeric material should cover several requirements such as: (i) biodegradability; (ii) biocompatibility; (iii) processability and suitable structure architecture, and (iv) sufficient mechanical properties [42].

(i) Biodegradability

Polymeric constructs used for flexor tendon repair are not intended to permanently remain in the human body. Therefore, the construct must be preferably biodegradable. Biodegradation of such polymeric constructs releases by-products that should be non-toxic and able to be absorbed by, and ultimately exit, the body through metabolic pathways. These by-products also may not interfere with other organs in the body [12,42,126]. The construct cannot lose its mechanical properties during the biodegradation prior to the complete healing of the tendon. Generally, flexor tendon injuries can take 9 to even up to 12 weeks to heal (maturation takes even longer up to 12 months) [49], which must be matched with the biodegradation rate. The biodegradation process is greatly influenced by the external environment where it takes place. Polymer degradation is initiated when it comes into contact with surrounding fluids inside the body. This degradation process then leads to the formation of lower molecular weight polymers, oligomers, and eventually monomers by chain scission [127].

Chemical degradation via hydrolysis (which can be enzyme-catalyzed) is possible on every degradable polymer due to the presence of hydrolyzable bonds [128,129]. The molar mass and the degree of crystallinity are proven to have a significant effect on the degradation rate of polymers and are therefore important parameters to consider when designing a certain construct. An increase in molecular weight results in an increase of scissions needed to degrade the material [130]. Similar results can be observed for the degree of crystallinity where an increase resulted in a decrease in the degradation rate. This can be attributed to the amorphous sections of the polymer which will degrade first [131,132].

Research has observed that mechanical loads have a significant accelerating effect on the material biodegradation rate. Observations were made that the biodegradation rate of poly(D, L-lactic acid) (PDLLA) increased while applying a continuous tensile load in comparison to no applied tensile load. In addition, the combination of both tensile and compressive loads had an even further increased effect on the biodegradation rate [133,134]. The mechanical loading should always be considered when the biodegradation rate of a biodegradable polymer is regulated.

(ii) Biocompatibility

Biocompatibility is essential when polymeric materials are used for any biomedical application. This means that the construct should have an appropriate response to the host for flexor tendon repair [135,136]. Other definitions describe biocompatibility as non-immunogenic, non-toxic, non-thrombogenic, and non-carcinogenic. In most cases, a cytotoxicity test is conducted to determine the biocompatibility of a material. Hereby, the effect of toxic agents derived from the polymeric material on cell viability and cells can be determined [137]. A polymeric material is believed to be cytotoxic when cell viability is <70%, measured during in vitro cell seeding tests [138].

(iii) Processability and Structure Architecture

Processability is another important requirement to get a construct commercially and clinically viable. The processing technique used to create the construct should be easy to scale up and more importantly, be cost-efficient. Polymers can be processed into films/sheets or tubular, nanofibrous membranes by electrospinning or directly injected in vivo [139]. It is important that the construct enables nutrient, growth factor, and cytokine permeation during tendon healing, which is possible due to the porosity of the membrane. In addition, porosity will allow permeation of the degradation by-products through the membrane so they can be metabolized by the body, avoiding high concentrations of by-products (acidic when polyesters are used) leading to cytotoxicity [19].

In addition, sufficient porosity must be achieved to be able to promote the formation of blood vessels. Finally, the construct should also resemble the native ECM as much as possible to avoid rejection of the body, which is an advantage of using nanofibrous membranes.

(iv) Mechanical Properties

When developing a construct, utilized for flexor tendon repair and healing, having good mechanical properties is an essential factor to take into consideration. Identification of the mechanical strength can be achieved by measuring the impact resistance of the final construct to maintain its integrity during implantation [140]. Tensile tests include the most common mechanical tests to evaluate a construct. During tissue remodeling, each application requires a different working range for mechanical properties as it is desirable that the construction resembles the mechanical properties of the native organs or tissue. For example, the flexor tendon has ultimate tensile strength (UTS) values between 2.98–3.98 MPa, in combination with an elongation at a break between 10–12% (Figure 2) [141], which is needed for immediate active mobilization after repair, during the healing process [142,143]. Several research projects, focusing on polymer-based constructs for flexor tendon repair, have tried to achieve these optimum mechanical properties but often did not succeed, Chen et al. [98] for example achieved a UTS of 1.43 ± 0.13 MPa. However, last year (2021) Pien et al. [93] managed to achieve the required mechanical strength due to the innovative solution of working with a multi-layer construct, in which the middle layer of polyethylene acts as a Chinese finger trap, increasing the mechanical strength. The biomechanical stability also depends on other factors such as degradation rate, absorption at the interface, and elasticity, which should be considered.

### 4.3. Materials for Flexor Tendon Scaffold and Construct Designs

**Figure 4:**
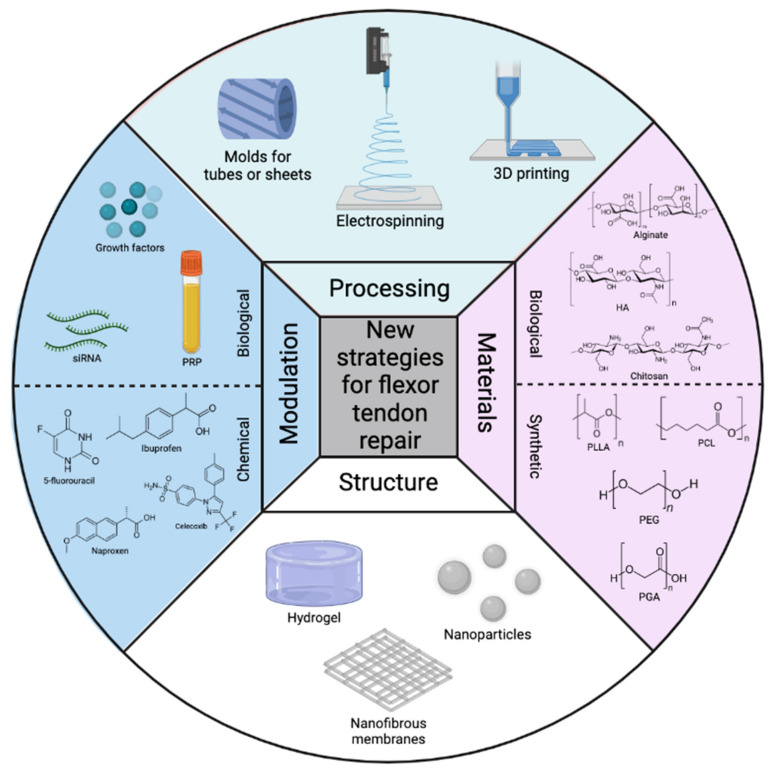
Overview of the most commonly used processing techniques, polymeric materials, structures, and modulations for flexor tendon repair in this review paper.

Polymeric materials used for flexor tendon repair can either be biological, synthetic (Figure 4), or semi-synthetic. Biological polymers have gained interest because of the therapeutic properties of the construct itself. Chitosan for example has both an anti-inflammatory as well as an antimicrobial response [19]. Problems such as toxicity and chronic immunological reactions, sometimes occurring with synthetic polymers, are frequently avoided using these biological materials [18,144]. However, synthetic polymers are widely understood in the field of biomedical applications, mainly for their mechanical properties and ease of processing into porous structures [145], compared to biological materials. Biological polymers do not have this wide variety of possible processing techniques, but their ability to mimic the native ECM and often better biocompatibility compared to synthetic polymers makes them very interesting [146]. Despite these advantages, biological polymers often have inferior mechanical properties [15] and (too) short biodegradation times compared to synthetic polymers. Finally, other major drawbacks of biological polymers are their high polydispersity, limited purity, and batch-to-batch molar mass variations [147] causing varying characteristics which is unacceptable from a biomedical point of view. Both polymer types have the ability to be functionalized by chemical and biological compounds, controlling their chemical, biological and physical properties [148].

Evaluation of recent literature shows that a combination of both polymer types can form a solution for current clinical complications in flexor tendon repair such as adhesion formation, lack of mechanical properties, and risk of infection. These are the so-called semi-synthetic materials, formed by the combination of biological and synthetic polymers using copolymerization, grafting, crosslinking, or blending. However, the incorporation of biological polymers in synthetic materials is also done in the literature. These are still classified as synthetic materials in this review paper as the biological compounds are solely released as drugs and are not added to strongly influence the properties of the construct (i.e., mechanical properties, biodegradation rate, etc.). In the case of semi-synthetic materials, the combination of both polymer types results in a superior polymer-based construct combining the desirable mechanical strength of synthetic polymers with the therapeutic and biocompatible properties of biological polymers [149], while maintaining a desired degradation rate.

This section is divided into three subsections based on the nature of the polymers. Each section also has an overview table of past research arranged according to the year of acceptance. This makes it possible to assess the evolution of these polymeric materials and to evaluate the advances in state-of-the-art development of flexor tendon repair using material-based constructs.

#### 4.3.1. Biological Polymer Constructs

Polymeric constructs of purely biological origin have been studied extensively. In the case of flexor tendon repair applications (Table 1), there is a stronger tendency to use protein-based materials, such as collagen, as it is predominately present in the native tendon tissue. Collagen was therefore one of the first biological polymers to be used for tendon recovery [147]. Silks can also be defined as protein polymers, consisting of fibroin and sericin [150]. These polymers are mostly derived from animals, although they are often in need of crosslinking in order to improve the mechanical strength, remove foreign antigens and possible diseases and decrease the degradation rate [151]. This is necessary, since a construct may not biodegrade or lose mechanical properties prior to 9–12 weeks of healing [49]. The increased use of these fibrous proteins can be attributed to their highly repetitive primary sequence, leading to an improved homogeneity in the secondary structure, i.e., β-sheets for most silks and triple helices in the case of collagen. Because of these repetitive structures, this family of protein polymers provides better mechanical properties compared to other biological materials, in combination with their biocompatibility and ability to tailor sequence by genetic control provides a basis to exploit these biological polymers for flexor tendon repair construct design [152].

Between 2013 and 2015, as seen in Table 1, research [153,154] was published where collagen membranes were derived from animals followed by sterilization, freeze-drying, and crosslinking to wrap around the injured tendon site after suturing. An increase in maximal tensile loading was observed compared to the traditional suturing technique. In addition, the membrane acted as a physical and mechanical barrier to significantly prevent adhesion formation. In 2015, collagen was crosslinked with glycosaminoglycan (GAG) into a resorbable matrix, which further reduced the adhesion formation. Despite these promising results, it was proven in 2016 by Wichelhaus et al. [155] that the use of collagen, in combination with elastin, cannot be advised for biological constructs as it enhances both extracellular and cellular inflammation. In addition, increased tendon gapping was observed as a negative result. These mixed results, in combination with a fast in vivo degradation directly accompanied by loss of mechanical strength [151], are believed to be the reason why the use of pure collagen was never researched further as a suitable primary material for tendon repair. However, a few more recent studies [156,157,158,159] reported the use of amniotic membranes in combination with protein-based polymers like collagen and silk for construct design, suppressing inflammation, and neovascularization.

The use of polysaccharides has recently increased for tendon repair, explained by the fact that these biological polymers often have therapeutic properties, actively helping the healing process. HA can be used for tendon repair with an additional anti-inflammatory effect by suppressing pro-inflammatory cytokines and chemokines and creating improved cellular proliferation response at the injury site, preventing adhesion formation as described in Table 1. It is the largest component of GAGs and is known to be non-immunogenic [160]. Nevertheless, HA has a very short in vivo degradation time of around 4 days by hyaluronidase [161]. Therefore, several chemical modifications and crosslinking options have been explored to decrease the degradation rate and improve its processability. Past research from Yilmaz et al. [162] and Isık et al. [163] described a platform where a HA membrane, combined with carboxymethylcellulose (CMC), was created in order to achieve a slow degradation in the body. CMC acts as a chemical carrier, used in various drug and food preparations [157]. Despite these promising results, the biodegradation time of HA is still too short for it to be a primary material for construct design. Embedding HA in a mildly cross-linked alginate hydrogel [164] increased the biodegradation time of HA from 4 to 11 days in vivo, which for the current application is still too short. Alginate is commonly derived from brown seaweed or bacteria and shows low toxicity and excellent biocompatibility [165]. In 2006, the use of a high concentration alginate solution was tested as a coating over the injured site [166]. CaCl_2_ was purposely not used as a hard gel would form, which would no longer be injectable. Animal studies on rabbits were performed, which were treated with the alginate solution, whereafter the adhesion formation was histologically and biomechanically tested. A higher flexion region and decrease in scar tissue formation were observed for rabbits treated with the alginate solution.

**Figure 5:**
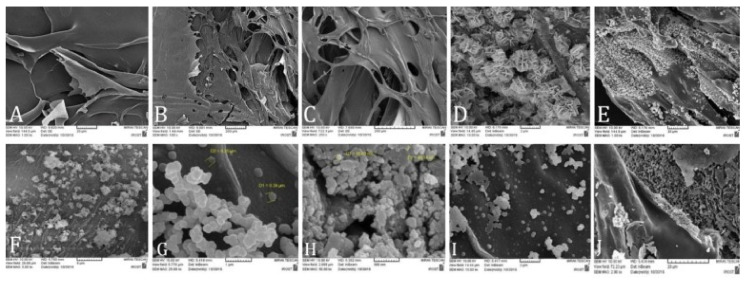
SEM ultra-micrographs of a tubular construct made from chitosan. (**A**–**C**): pure chitosan, (**D**–**F**): ZnO coated chitosan and (**G**–**J**): ZnO nanoparticles coated chitosan. Reprinted with permission from ref. [173]. 2018 A. Yousefi.

Another polymer in the list of polysaccharides with therapeutic properties is chitosan. These properties include cell proliferation, fibroblast growth inhibition, anti-microbial response, and induced collagen production [167,168,169]. Chitosan, which is cationic and commonly derived from shrimp shells [170], is often combined with alginate or HA due to their opposite charges to form poly-ionic complexes. Chitosan can be produced into an injectable gel that is capable of reducing adhesion formation [169]. The effect of chitosan on the sirtuin (SIRT)1 protein expression, responsible for the inhibition of inflammation in human tenocytes [171], was also investigated and proved to be elevated when chitosan was injected. In addition, a higher maximum tensile loading was observed when the flexor tendons were treated with chitosan injections. The use of a thermo-responsive in-situ forming hydrogel, based on poly(N-isopropylacrylamide) containing chitosan and HA, was also reported in 2017 [172]. A sol-gel transition of the injectable hydrogel was observed at a lower critical solution temperature (LCST) of 31.4 °C. One year later, Yousefi et al. [173] incorporated zinc oxide nanoparticles into a novel tubular construct made from a chitosan solution in a mold after freeze-drying (see Figure 5). Literature proved that zinc has an antimicrobial response and is an essential micronutrient for metabolism, catalyzing more than one hundred enzymes [174,175,176].

The degradation time of biological polymers is strongly dependent on their molar mass, which is no exception for chitosan. Commonly, chitosan shows fast partial degradation within the first 10 days whereafter it slows down and is fully degraded after 1–2 months [177]. For this reason, similar to HA, it is not possible to use chitosan as the main component of a polymeric construct for tendon repair.

These polysaccharides are often processed into hydrogels sheets or membranes (Figure 4) using various techniques such as crosslinking, grafting, ionic interactions, hydrogen bonding, etc. The use of hydrogels also allows for easy injection at the injured site to act as a barrier against adhesion formation combined with an increased residence stability time of the therapeutic agent in the body [178]. However, a major drawback of hydrogels for flexor tendon repair is their ability to absorb large amounts of water, causing the construct to strongly swell which puts pressure on surrounding tissue and decreases mechanical properties, therefore decreasing the healing capability [179]. For this reason, polysaccharides like HA, alginate, and chitosan have found more applications in wound healing and as drug delivery systems when loading as an active compound inside another polymer material due to their proven anti-inflammatory effect [14,19,93,106,180]. In addition, polysaccharides are commonly combined with synthetic polymers into semi-synthetic polymers.

#### 4.3.2. Synthetic Polymer Constructs

In comparison to the evolution of platforms using biological polymers for flexor tendon construct design, the same trend, being that initially several constructs were evaluated made from one pure polymer, was observed already earlier in the use of synthetic polymers as biomedical construct designs as shown in Table 2. Each of these individual polymers has certain shortcomings whereafter it was observed that the mechanical properties, in addition to the degradation kinetics and their interactions with the surrounding tissue, could be tailored by blending or copolymerization [126].

Aliphatic polyesters including, poly(ε-caprolactone) (PCL), poly(L-lactic acid) (PLLA), and poly(glycolic acid) (PGA) (Figure 4) have been attractive for flexor tendon repair constructs due to their biodegradability by mostly non-enzymatic hydrolysis of the ester backbone into acidic by-products. Notice that these could cause an immunological reaction with the surrounding tissue at the repair site [12]. Synthesis of these aliphatic polyesters is commonly performed by ring-opening polymerization starting from the cyclic dimers [181]. These polyesters are commonly processed into fibers by i.e., electrospinning [97,105,108], melt electrowriting [182,183], or 3D printing [184,185] (Figure 4) to mimic the native ECM of the flexor tendon and are among the few synthetic polymers which have been approved by the FDA in several clinical applications since the 1970s [186].

Up until 2013, mostly biological polymers were used for flexor tendon repair constructs. A shift to more synthetic polymer-based constructs can be observed in the last decade and is described in Table 2. Materials like PLLA and PCL were among the first to be processed into nanofibrous membranes by electrospinning due to their relatively high toughness and mechanical strength [131]. In addition, PLLA and PCL are hydrophobic, meaning the swelling of the construct is minimal. The first paper describing the use of the synthetic polymer PLLA in construct design for flexor tendon repair was published in 2013 [187]. In this research, dextran glassy nanoparticles (DGNs) were used as a carrier for loading bFGFs. Hereafter, the loaded DGNs were blended with PLLA, and a fibrous membrane was produced by electrospinning, capable of sustainably releasing bFGFs. They highlighted that the use of the PLLA membrane resulted in enhanced cell proliferation and intrinsic tendon healing.

In the same year, the first papers were published that loaded ibuprofen into a nanofibrous membrane constructs based on synthetic polymers [94,95,96]. The membranes achieved improved biological outcomes (Table 2) compared to similar research due to the use of ibuprofen, which is a non-steroidal anti-inflammatory drug capable of reducing pain and improving the healing process. Additionally, ibuprofen is proven to have an antipyretic response in vivo. As opposed to its beneficial properties, higher concentrations of ibuprofen can lead to increased cytotoxicity, as was proven by Shalumon et al. [123]. From 2015 to 2021, four papers have been published capable of solving the risk of a cytotoxic ibuprofen burst release, of which two contained synthetic materials [97,188] (Table 2) and two semi-synthetic materials [98,123] (see further Section 4.3.3).

PLLA has a relatively long degradation period of approximately 6 months depending on the environment, accompanied by the acidic biodegradation by-products, which may be responsible for a prolonged risk of inflammation. The degradation period can be shortened by copolymerization with poly(ethylene glycol) (PEG) [95]. It is important to notice that PEG is hydrophilic, increasing the overall hydrophilicity of the construct, making it more prone to swelling. Jiang et al. [101] believed in 2014 that the peritendinous adhesion formation may be due to the proliferation of fibroblasts and excessive collagen synthesis. Therefore, they looked more into the Extracellular Signal-Regulated Kinases 1 and 2 (ERK1/2) and Small Mothers Against Decapentaplegic 2 and 3 (SMAD2/3) pathways responsible for this. A hypothesis was made that the proliferation of fibroblasts and excessive collagen synthesis could be inhibited by downregulating ERK1/2 and SMAD2/3 phosphorylation of exogenous fibroblasts by using celecoxib. Therefore, celecoxib was incorporated into a PELA fibrous membrane, processed by the commonly used electrospinning technique. Identical to the use of ibuprofen, an excellent continuous celecoxib release pattern was observed. Their hypothesis was confirmed by the reduced type I and type III collagen expression. In 2021, celecoxib was also used in a PCL electrospun nanofibrous membrane for the treatment of failed back surgery syndrome with similar results of anti-inflammatory response [189].

Regarding the evaluation of the complications occurring at flexor tendon operative measures, in the beginning, only adhesion formation was researched. Solutions for infections by microbials were at that time not yet incorporated into the constructs. As mentioned in Section 4.1.2, infections are caused by bacteria entering the repair site during operations or healing. In the literature, Ag NPs are commonly used as clinically approved material for inducing an antimicrobial response in the body [68,94,104,108,123,124,125,190]. They are highly effective against pathogens responsible for an infection such as *S. aureus* and *E. coli*, with the absence of inducing drug resistance. Their ability to inactivate enzymes and proteins located in the cell membrane blocks cellular functionally. In addition, internal wound and skin healing, often accompanied by tendon injuries, is promoted by reducing the activity of matrix metalloproteinases. Finally, Ag NPs are responsible for the decrease in K^+^ ions, which results in the damaging of bacterial cell membranes and ultimately cellular death [191,192]. However, there still are some concerns about the use of Ag NPs, which may lead to liver damage and kidney failure. The group from Lui et al. [94] combined both the anti-inflammatory effect of ibuprofen and the antimicrobial effect of the Ag NPs into a PLLA fibrous membrane construct. The construct was capable of reducing both, most impacting, complications occurring during flexor tendon healing. However, the antimicrobial effect was only tested in vitro, therefore further in vivo tests are still required.

In 2015, a paper was published that described the use of PCL instead of PLLA as a construction material [193]. PCL, a semi-crystalline polymer, also widely used in biomedical applications, is compatible with both soft and hard tissue applications [194]. It is always present in its rubbery state due to the low glass-transition temperature of ~−60 °C, with a degradation time of up to 4 years. Therefore, constructs produced solely from PCL have limited applications. PCL, similar to PLLA, has a very slow in vivo degradation rate, much lower compared to other synthetic polyesters, and is thus commonly combined with PGA or PEG to increase the degradation rate and decrease its brittle behavior. Therefore, a blend of PCL with different weight fractions of PEG was processed into a monolayer nanofibrous membrane using the same techniques mentioned above, where altering the PEG wt% influenced several mechanical and biological properties [193]. The most important observations made were that an increase in PEG wt% resulted in a lower UTS, faster biodegradation, and a reduced adhesion formation. Therefore, it is important to compare these properties when opting to choose possible blending combinations. Another study from 2020 combined the advantages of a PCL nanofibrous membrane with the slow release of bFGFs inside an amnionic membrane into a multi-layered construct [195]. The adhesion and proliferation of tenocytes and fibroblasts and collagen synthesis were enhanced by up-regulating the phosphorylation of ERK1/2 and SMAD2/3, which was contradictory to previous research [101]. In addition, the multi-layer nanofibrous membrane effectively isolated the exogenous adhesion tissue and promoted endogenous tendon healing.

Synthetic polymers, used for flexor tendon repair, are not always processed by electrospinning. Wu et al. [103] and Yuan et al. [99] developed and processed a block copolymer of PCL and PEG loaded with dexamethasone micelles and a block copolymer of PLGA and PEG loaded with 5-fluorouracil respectively into thermo-responsive injectable hydrogels. These hydrogels showed promising results in reducing the adhesion formation through a controlled release of dexamethasone as anti-inflammatory glucocorticoids [196] and 5-fluorouracil as a non-steroidal anti-inflammatory drug. Despite the promising results of utilizing hydrogels for flexor tendon repair, they commonly biodegrade in less than 28 days, which is too short for a complete recovery of the flexor tendon. Additionally, as mentioned before, they are able of absorbing large amounts of water, which causes swelling of the construct. Hydrogels are mostly injected at the healing site that has been repaired by a traditional technique such as suture, to achieve a more superior and controlled healing process.

**Figure 6:**
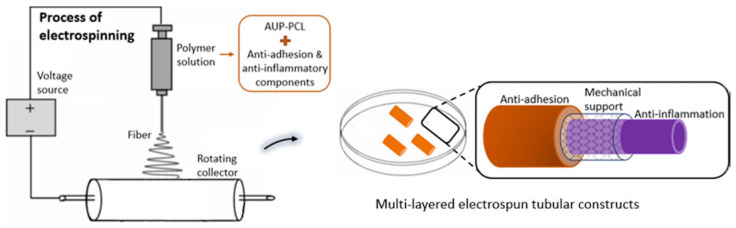
A Multi-layered tubular construct from a novel material, acrylate endcapped urethane-based precursor (AUP) with a PCL backbone as the outer and the inner layer, in combination with HA as anti-adhesion and naproxen as an anti-inflammatory. The polypropylene braided structure in the middle acts as mechanical support, based on the Chinese finger trap mechanism. Reprinted with permission from ref. [89]. 2021 N. Pien.

Pien et al. [93,105] published the most recent research papers (2021) on processing synthetic polymer-based materials for flexor tendon repair into a multi-layered tubular construct (Figure 6) instead of sheath membranes. A novel acrylate endcapped urethane-based precursor (AUP) with a PCL backbone was used in combination with HA as anti-adhesion and naproxen as an anti-inflammatory compound respectively to create an outer and inner layer, by electrospinning. The construct is classified as synthetic because HA was added for the sole purpose of a drug and did not strongly influence the mechanical properties of the construct. Naproxen has a similar mechanism in the body compared to ibuprofen by reducing inflammation and pain due to reducing the responsible hormones. However, naproxen has a longer-lasting effect as proven by literature [197,198,199]. Often, the mechanical properties of the construct did not suffice for optimal healing of the tendon by active mobilization. Therefore, the middle layer was composed of a polypropylene braided structure, based on the mechanism of a Chinese finger trap, acting as a mechanical support and significantly enhancing the mechanical properties measured by uniaxial tensile tests.

Additionally, the construct had been functionalized with methyl acrylate groups, which gives the AUP the possibility to form strong covalent bonds due to UV-crosslinking after electrospinning, therefore creating extra interactions between the inner and surrounding layers. In a further study [105], the tubular construct was evaluated using a rabbit model, proving its anti-adhesion response and sufficient mechanical properties for tendon repair and healing. A next step could still be to include antimicrobial compounds to counter infections. In recent years, apart from the constructs mentioned above, there has been a transition from synthetic, more towards semi-synthetic polymer-based constructs as explained in Section 4.3.3.

#### 4.3.3. Semi-Synthetic Polymer Constructs

The use of semi-synthetic materials, combining biological and synthetic polymers, to design a polymer-based construct in flexor tendon repair has become more popular. A clear transition from synthetic to semi-synthetic polymer-based constructs is observed in the literature, starting from 2014 shown in Table 3. This can be explained by the fact that the therapeutic benefits of biological polymers were further researched around this time.

A study is mentioned in Section 4.3.2 details the preparation of a synthetic nanofibrous membrane made from electrospun ibuprofen-loaded poly(l-lactic acid)-poly(ethylene glycol) diblock copolymer. Indeed, literature expects that membranes made from polymer blends or copolymers enhance the anti-adhesion response compared to homopolymer membranes. However, the production of such copolymers includes complex steps. Additionally, extreme care is required in controlling the electrospinning conditions. An alternative for blends or copolymerization is to produce a surface-grafted membrane. As mentioned in Section 4.3, the synthesis of copolymers by grafting is one of the techniques for producing semi-synthetic materials. The grafting takes place after electrospinning, which allows for more flexibility in choosing the grafting material and in the electrospinning conditions. Often polysaccharides such as chitosan and HA, due to their therapeutic properties, are surface-grafted onto a polyester backbone [19,107,180]. Surface grafting of the anti-inflammatory polymers could provide a sustained release over several weeks during biodegradation. In 2014, Chen et al. [107] was the first in the literature to report the use of surface grafting chitosan to an electrospun PCL membrane using plasma. Surface grafting of chitosan on PCL had no effect on the fiber diameter, morphology, microstructure, and permeability, while at the same time improving its mechanical properties and reducing fibroblast attachment. Literature also reports the surface grafting of another polysaccharide such as HA onto PCL, which proved to have the same results compared to the chitosan grafting without the antimicrobial effect [107]. An increase in UTS was observed when HA was grafted on PCL which was a promising result as no trade-off had to be considered between the anti-inflammatory response and the UTS compared to other research in the same year [106] where a core-shell nanofibrous membrane of HA/PCL was produced. Hereby, the conclusion can be made that surface grafting of the chitosan and HA to PCL allows for a more sustainable and accurate anti-inflammation response at the injured flexor tendon site. Both membranes showed a significant decrease in peritendinous adhesion formation. In 2018, a paper was published describing the same plasma technique for surface grafting of HA, however, poly(lactide-co-ε-caprolactone) (PLCL) was used as the backbone instead of pure PCL [180]. The biodegradation period was slightly decreased due to the copolymerization, although the overall anti-inflammatory response remained similar.

In the last decade, another platform to produce a dual functional semi-synthetic polymer-based construct has been investigated. The platform describes the use of core-shell nanofibers by co-axial electrospinning. Several papers, starting from 2012 up until 2021 have been published using this core-shell co-axial electrospinning technique [100,104,106,108,123,200]. The core commonly consists of a therapeutic agent such as HA and the shell is made from polyester such as PCL, PLA, PELA, etc. Such a construction provides both the strong mechanical properties of the polyester and the slow release of HA for an anti-inflammation effect and improved tendon gliding. However, this processing technique requires much more complex electrospinning conditions compared to the previous surface-grafting technique. In addition, the HA core material is responsible for a decreased UTS compared to the pure polyester fibers. A significant advantage of the co-axial electrospinning technique is the possibility to load a different chemical/biological compound in the outer shell, compared to the inner core. Several papers report embedding Ag NPs into the outer shell of the fiber, therefore considering tackling the risk of infection during the operation or healing period [104,108,123]. The Ag NPs showed no cytotoxicity for all constructs. Another study, published in 2016, used the same platform and embedded celecoxib into the core which was responsible for a decreased expression of type I and type III collagen and cell proliferation [100]. A paper published in 2021 [108] investigated the difference between a thick and thin shell. The release rate of HA from the core could be controlled by varying the size of the shell to achieve the optimal anti-adhesion effect. The thin shell fibers demonstrated the optimal outcomes with limited cytotoxicity of the Ag NPs. The same year, another interesting state-of-the-art study [200] using core-shell fibers investigated the influence of the combined effect of fiber orientation and platelet-rich plasma (PRP) on tendon healing. The core of the fibers was loaded with PRP, which is a blood plasma fraction with platelet-rich cellular components and plays a key role during tendon healing and regeneration [201,202]. The sustained release of growth factors from PRP provided a biochemical stimulus for tendon healing. Another study described the function of aligned fibers (mimicking the physiological environment of the tendon). This alignment of the fibers improved cell proliferation compared to randomly oriented fibers.

**Figure 7:**
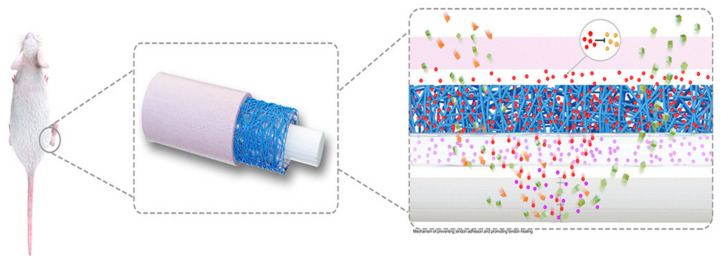
Double layer composite membrane with electrospun nanofibrous poly(lactic-co-glycolic) ibuprofen-loaded inner layer and a hydrogel of poly(ethylene glycol)-block-poly(L-valine) as an outer layer for preventing tendon adhesion and promoting tendon healing. The hydrogel coating prolonged the ibuprofen release in vivo. Reprinted with permission from ref. [97]. 2021 Z. Yan.

As mentioned in Section 4.3.2, a multi-layer tubular construct was produced by Pien et al. [93,105] in 2021. However, this was not the first published paper reporting the use of a multi-layered construct for flexor tendon repair. In 2015, a multi-layer semi-synthetic polymer-based construct was produced with celecoxib-loaded PELA electrospun fibrous membrane as the outer layer, HA hydrogel as the middle layer, and PELA electrospun fibrous membrane as the inner layer by sequential electrospinning [102]. The inner layer of PELA and HA was proven to mimic the biological response of HA secretion in order to enhance tendon gliding and tendon healing, while the outer celecoxib-loaded layer reduced the adhesion formation. A similar approach was taken by Deepthi et al. [109] one year later using electrospun PLLA, to mimic the aligned collagen fibers, blended with collagen-chitosan hydrogel in order to mimic the glycosaminoglycans of sheath ECM for tendon regeneration. Thereafter, the fibers were coated with alginate for a superior healing process. The construct achieved the same promising results without the suppression of collagen expression by inhibition of ERK1/2 and SMAD2/3 phosphorylation by celecoxib. A double layer composite membrane with electrospun nanofibrous poly(lactic-co-glycolic acid) (PLGA) inner layer and a hydrogel of poly(ethylene glycol)-block-poly(L-valine) as outer layer was produced in 2021 [97] and is shown in Figure 7. The addition of the outer hydrogel layer was responsible for a sustained release of ibuprofen over a period of 34 days in vitro without a burst release. Therefore, higher concentrations of ibuprofen could be loaded, increasing the anti-inflammatory effect of the construct.

In 2019, different materials were used [98] in comparison to the commonly used aliphatic polyesters and PEG. HA loaded with ibuprofen was processed into a nanofibrous membrane by electrospinning, which was similar to previous research, but was followed by dual ionic crosslinking with FeCl_3_ and covalently crosslinked with 1,4-butanediol diglyceryl ether (BDDE). The study aimed to use iron ions (Fe^3+^) and BDDE as a crosslinking agent to achieve a prolonged release of ibuprofen. Higher concentrations of ibuprofen could be used without inducing cytotoxicity and improving the mechanical properties. However, ibuprofen loading above 30% induced cytotoxicity, tested by in vivo rabbit models. An innovating approach was published in [203] in the same year. They developed a promising platform using gene silencing in reducing the risk of peritendinous adhesion formation. In recent years, gene silencing via the delivery of small interfering RNA (siRNA) has experienced rapid advancement in the downregulation of specific genes [204]. As such, SiRNA therapies have been developed in the most recent years using viral or non-viral vectors, mainly involving peptide conjugation [205], cationic polymers [206], and liposomes [207]. Unfortunately, a bottleneck has gradually emerged in the siRNA technology around the transient silencing effect [208]. Therefore, an urgent need was created for a sustained release of siRNA via carriers, followed by a consequent increase in bioavailability. In this study, a cationic polymer, 2,6-pyridinedicarboxaldehyde-polyethylenimine (PDA), was used as a delivery system for ERK2-siRNA in combination with electrospun PLLA/HA fibers. Silencing ERK2 can indirectly inhibit type I and III collagen production and directly inhibit fibroblast proliferation as mentioned before [101]. A sustainable, cumulative release of 80% ERK2-siRNA in 30 days was achieved. Inhibition of cell proliferation showed that the membrane could retain the bioavailability of ERK2-siRNA, mostly due to PDA.

## 5. Conclusions and Perspectives

Flexor tendon injuries are common and mostly treated operationally. Hereby, several postoperative complications may occur such as infection, wear, tendon scar tissue formation, mechanical failure, and excessive adhesion formation. Multiple therapeutic reconstruction techniques, such as grafting or suturing, have been used in the past, but they don’t offer an adequate long-term solution. Current strategies for preventing the formation of peritendinous adhesion rely mainly on the inhibition of inflammation, using physical/mechanical barriers for averting wound contact with surrounding tissue, downregulating the ERK1/2 and SMAD2/3 phosphorylation, and inhibiting the excessive type I/III collagen and proliferation of fibroblasts. The use of polymer-based constructs can play a variety of important roles in preventing the formation of adhesion, not only by forming a physical barrier. Most current researchers focusing on flexor tendon repair develop a construct that is equipped with both anti-inflammatory as well as antimicrobial agents, solving the most severe postoperative complications occurring during tendon healing. The use of only synthetic or biological polymers is not sufficient for creating a multi-functional construct, therefore functional materials and combinations of several polymer types (semi-synthetic) have been engineered in combination with drugs, which are as a result highly effective in preventing these postoperative complications. This review provided a comprehensive summary of the mechanism of tendon injuries and healing. In addition, it contains a compilation of constructs for tendon repair found in the literature for each different polymer type (biological, synthetic, and semi-synthetic). Besides the material type, several techniques, structures, and even the incorporation of chemical and biological drugs have attracted more attention recently and were included in the review. These new approaches in the development of new constructs are likely to result in an enhanced healing treatment for flexor tendon injuries. At present, a lot of these constructs already show great in vivo results in animal models, mostly mice or rabbits, although only limited studies have been performed on humans. The fast pace of development in the field will undoubtedly lead to the use of smart materials and multi-functional constructs, used in clinical practice. Although excellent results were obtained from past research, precise engineered constructs and the development of new drugs are not yet at their peak performance and the field of flexor tendon repair still requires new approaches and techniques.

Constructs can contain multiple drugs or biological compounds and fulfill combination treatment where the active payload release could be controlled in the future depending on the healing stage of the tendon. Hence, structures can heal the damaged or lacerated tendon at the first stage and later help in the maturation period of tendon healing. Active control of the drug release could also avoid commonly observed burst releases and avoid the accompanying possible side effects. Nano-based drug delivery has already proven to be successful in other medical fields to eradicate the problem of a burst release [209]. Future work may include the incorporation of nanoparticles into the nanofibrous constructs, i.e., electrospinning. The controlled active drug release can be accomplished in the future by engineering a smart polymeric construct that could have a trigger based on temperature, pH, electrical signals, magnetic field, and many more. Although such smart triggers have already been incorporated into other biomedical applications such as tumor immunotherapy [210], temperature-dependent drug and gene delivery [211], neural tissue engineering [212], skin wound healing [213], and many more, it is not yet developed for flexor tendon repair constructs.

## Figures and Tables

**Table 1:** Compilation of publications showing the evolution of biological polymer-based constructs intended to treat flexor tendon injuries.

Year	Material and Structure	Material Processing Technique	Mechanical Response ^1^	Biological Response ^1^	Ref.
1999	Membrane of HA	Crosslinking	-	Decrease in adhesion formation	[163]
2006	Coating of ALG solution	Sodium ALG derived from Lassonia nigrescens, non-crosslinked and sterilized	Higher flexion region	Decrease in scar tissue formation Tendon healing with longitudinal remodeling compared to random remodeling in control	[166]
2010	Membrane of HA	-	Increased UTS	Decrease in adhesion formation	[162]
2012	Hydrogel of mildly crosslinked ALG embedded with HA (HA/mcALG)	Crosslinking	Sustained release of HA	Slower degradation rate of HA/mcALG compared to the pure HA hydrogel Decrease in adhesion formation for both HA as well as HA/mcALG constructs	[164]
2013	Membrane of collagen	Bovine derived, freeze-dried, and sterilized	Increased UTS after 8 weeks	Decrease in adhesion formation	[153]
2015	Membrane of collagen	Porcine derived, sterilized, and rehydrated	-	Decrease in adhesion formation for both the collagen membrane and collagen-GAG matrix sheet	[154]
	Porous matrix sheet of Collagen-GAG	Crosslinked and rehydrated			
2015	Hydrogel of CHI	-	Increased UTS	Decrease in adhesion formation CHI-induced SIRT1 protein expression	[169]
2016	Membrane of collagen/elastin	Matriderm^®^	Increased gapping	Decrease in adhesion formation	[155]
2016	Amnionic membrane combined with silk	Sericin removed by solution, soaked in 1% collagen/HA, and dried, freeze-dried, and sterilized. Amnionic membranes harvested from human placenta	Increased UTS	No immigration of inflammatory cells or fibroblast-like cells Formation of new blood vessels	[156]
2017	Thermoresponsive hydrogel of HA-CHI-PNIPAm	Copolymer by grafting CHI on PNIPAm whereafter dissolved in HA solution	Sol-gel in-situ transition at an LCST of 31.4 °C	Decrease in adhesion formation Decrease in fibroblast migration Limited cytotoxicity	[172]
2018	Tubular construct of CHI with zinc oxide nanoparticles	Mold with CHI solution containing ZnO NP followed by freeze-drying	Complete biodegradation in 8 weeks	Decrease in adhesion Improved gliding Improved collagen synthesis due to Zn	[173]
2021	Hydrogel of HA embedded with rhynchophylline	Crosslinking	Sustained release of rhynchophylline Increased healing strength	Decrease in adhesion formation Increased expression of type I and III collagen Increased gliding excursion	[178]

^1^ Compared to traditional suture technique control. Abbreviations: Polymers: hyaluronic acid (HA), alginate (ALG), glycosaminoglycan (GAG), chitosan (CHI), poly(N-isopropylacrylamide) (PNIPAm). Other: ultimate tensile strength (UTS), sirtuin 1 (SIRT1), lower critical solution temperature (LCST) zinc oxide nanoparticles (ZnO NP).

**Table 2:** Compilation of publications showing the evolution of synthetic polymer-based constructs intended to treat flexor tendon injuries.

Year	Material and Structure	Chemical/Biological Modulation and Concentration	Material Processing Technique	Mechanical Response ^1^	Biological Response ^1^	Ref.
2013	NFM of PLLA	bFGFs loaded in DGNs	Electrospinning	Blending of DGNs decreased UTS and maximum elongation	Improved cell proliferation Improved intrinsic tendon healing	[187]
2013	NFM of PLLA-b-PELA	IBU	Electrospinning	-	Idem [187] Decrease in inflammation and adhesion formation	[95]
2013	NFM of PLLA–MMS	IBU	Electrospinning	Controlled release of IBU, without initial burst	Idem [95]	[96]
2014	NFM of PLLA-c-PELA	Celecoxib	Electrospinning	Controlled release of celecoxib	Idem [95] Decreased expression of type I and type III collagen	[101]
2014	NFM of PLLA	IBU + Ag NPs	Electrospinning	Controlled release of IBU, without initial burst	Idem [95] Antimicrobial response	[94]
2015	NFM of PCL/PEG blend (0, 25, 50 and 75 wt% PEG)	-	Electrospinning	Increasing wt% PEG decreased UTS and maximum elongation	Decrease in adhesion formation Higher PEG wt% led to decreased fibroblast attachment Good permeability for nutrients, growth factors, and cytokines	[193]
2015	Hydrogel of PLGA-PEG-PLGA	5-Fluorouracil	-	Sol-gel phase transition depending on temperature	Decrease in adhesion formation Full in vivo degradation in 28 days	[99]
2015	Hydrogel of PEG-b-PLC-b-PEG	DEX micelles	PEG-b-PLC-b-PEG dissolved in saline at 4 °C	Sol-gel phase transition depending on temperature	Decrease in adhesion formation Low cytotoxicity of hydrogel and micelles Full in vivo degradation in 20 days	[103]
2017	NFM of PLGA	IBU	Electrospinning of PCL Amniotic membrane was freeze-dried and decellularized	Increased UTS Sustainable release of IBU	Decrease in adhesion formation Inhibition of fibrosis via the COX2 pathway Reduced pain and neurological deficits	[188]
2020	MNFM of an amniotic membrane between two layers of PCL	bFGFs	Electrospinning	Increased work of flexion Decreased UTS	Increased phosphorylation of ERK1/2 and SMAD2/3 Enhanced collagen synthesis	[195]
2021	MNFM of PCL/AUP outer layer and inner layer, with braided monofilament PE as middle layer	Naproxen and HA loaded in PCL/AUP	Electrospinning	Significant increase in UTS No degradation prior to 9 weeks	Decrease in adhesion formation naproxen introduced no cytotoxicity	[93,105]

^1^ Compared to traditional suture technique control. Abbreviations: Structures: nanofibrous membrane (NFM), multi-layer nanofibrous membrane (MNFM). Polymers: poly(L-lactic acid) (PLLA), poly(L-lactic acid)-co-poly(ethylene glycol) (PELA), poly(ε-caprolactone) (PCL), poly(ethylene glycol) (PEG), poly(lactic-co-glycolic acid (PLGA). Modulations: basic fibroblast growth factors (bFGFs), ibuprofen (IBU), silver nanoparticles (Ag NPs), dexamethasone (DEX). Other: dextran glassy nanoparticles (DGNs), modified mesoporous silica (MMS), ultimate tensile strength (UTS), cyclooxygenase-2 (COX2), acrylate endcapped urethane-based precursor (AUP).

**Table 3:** Compilation of publications showing the evolution of semi-synthetic polymer-based constructs intended to treat flexor tendon injuries.

Year	Material and Structure	Chemical/Biological Modulation and Concentration	Material Processing Technique	Mechanical Response ^1^	Biological Response ^1^	Ref.
2012	CSNFM of PCL shell with a HA/PCL core	HA	Sequential and microgel electrospinning	Increasing wt% HA decreased UTS	Decrease in adhesion formation Increased wt% HA resulted in higher cell viability and cell proliferation after 1 day in culture	[106]
2014	NFM of PCL-sg-CHI	CHI	Electrospinning	Increased UTS for healed flexor tendons treated with the PCL-g-CHI membrane compared to Seprafilm and PCL membrane	Decrease in adhesion formation CHI showed no cytotoxicity	[19]
2014	NFM PCL-sg-HA	HA	Electrospinning	Increased UTS, maximum elongation, and Young’s modulus for PCL-g-HA compared to PCL	Decrease in adhesion formation Unaffected cell proliferation	[107]
2015	CSNFM of PCL shell with a HA core	Ag NPs + HA	Co-axial electrospinning	Decreased pull-out force for HA/PCL+Ag NPs Increased UTS for healed tendons treated with HA/PCL+Ag NPs compared to PCL membrane	Decrease in adhesion formation Ag NPs as an antimicrobial effect without significant cytotoxicity	[104]
2015	MNFM of PELA outer layer, HA middle layer, and PELA inner layer	HA + Celecoxib	Sequential electrospinning	Lower work of flexion	Decrease in adhesion formation Good permeability for nutrients, growth factors, and cytokines	[102]
2016	NFM of PLLA blended with collagen/CHI hydrogel, coated with ALG	CHI + ALG	Microgel electrospinning and solution coating	-	Decrease in adhesion formation Promotion of tendon gliding	[109]
2016	CSNFM with celecoxib loaded PELA shell with a HA/PELA core	Celecoxib + HA	Microgel and sequential electrospinning	No decrease in mechanical properties due to the use of celecoxib	Decrease in adhesion formation Promotion of tendon gliding Decreased expression of type I and type III collagen Decrease in cell proliferation	[100]
2018	CSNFM of PCL/PEG shell with HA core	HA embedded with IBU and PCL/PEG loaded with Ag NPs	Co-axial electrospinning	Elongation at break decreased and Young’s modulus and UTS increased for higher IBU wt% IBU showed fast release during first 8 h, but slows down over time	Decrease in adhesion formation Reduced fibroblast attachment and proliferation Higher IBU concentrations lead to substantial cytotoxicity in vitro and in vivo Promotion of tendon gliding Ag NPs as an antimicrobial effect without additional cytotoxicity	[123]
2018	NFM of PLCL-sg-HA	HA	Electrospinning	Decreased UTS Release rate of HA was controlled by sheath thickness	Decrease in adhesion formation Prevention of penetrating fibroblasts	[180]
2019	NFM of HA	IBU	Electrospinning followed by covalently crosslinking to BDDE and ionic crosslinking to FeCl3	Higher IBU loading increased UTS and Young’s modulus	Decrease in adhesion formation Prevention of cell attachment and penetration	[98]
2019	NFM of PLLA-HA	PDA loaded with ERK2-siRNA + HA	Electrospinning	Cumulative release of 80% ERK2-siRNA in 30 days	Decreased expression of type I and type III collagen Decrease in cell proliferation	[203]
2021	CSNFM of PLA shell with HA core	PLA loaded with Ag NPs + HA	Co-axial electrospinning	Sustainable release of HA	Best decrease in adhesion formation observed for thin shell fibers Ag NPs as an antimicrobial effect	[108]
2021	CSNFM of PCL shell with HA core	HA loaded with PRP	Co-axial electrospinning	Controlled release of proteins from PRP	Aligned fibers provided optimal cell proliferation Aligned fibers provided increase in type I and decrease in type III collagen Ag NPs as an antimicrobial effect without additional cytotoxicity	[200]
2021	NFM of PLGA coated with PEG-PLV hydrogel	IBU + bFGFs	Electrospinning followed by hydrogel coating	No obvious effect on tendon mechanical properties	Decrease in adhesion formation Increased expression of type I and III collagen	[97]

^1^ Compared to traditional suture technique control. Abbreviations: Structures: core-shell nanofibrous membrane (CSNFM), nanofibrous membrane (NFM), multi-layer nanofibrous membrane (MNFM), surface graft (sg). Polymers: poly(ε-caprolactone) (PCL), hyaluronic acid (HA), chitosan (CHI), poly(L-lactic acid)-poly(ethylene glycol) (PELA), poly(L-lactic acid) (PLLA), alginate (ALG), poly(ethylene glycol) (PEG), poly(L-valine) (PLV). Modulations: silver nanoparticles (Ag NPs), ibuprofen (IBU), 2,6-pyridinedicarboxaldehyde-polyethylenimine (PDA), extracellular signal-regulated kinase 2 (ERK2), small interfering RNA (siRNA), 1,4-butanediol diglyceryl ether (BDDE), platelet-rich plasma (PRP). Other: ultimate tensile strength (UTS).

## Data Availability

Not applicable.
